# Malt Extract—A Therapeutic Study

**Published:** 1891-10

**Authors:** John Aulde

**Affiliations:** Philadelphia, Penn.; Member of the American Medical Association, of the Medical Society of the State of Pennsylvania, of the Philadelphia County Medical Society, etc., etc.


### MALT EXTRACT—A THERAPEUTIC STUDY.

BY JOHN AULDE, M. D., PHILADELPHIA, PENN.

Member of the American Medical Association, of the Medical Society of the State of Pennsylvania, of the Philadelphia County Medical Society, etc., etc.

Within the past few years the general practitioner must have been forcibly impressed by the wide-spread prevalence of ailments, having their origin in the derangements of the digestive apparatus. Close observers have also witnessed numerous attempts on the part of manufacturing chemists to supply the medical profession with remedial agents to meet the demands of patients who sought relief. The last decade may, with propriety, be called the pepsin campaign. Physiological remedies have received such enthusiastic praise that we have been seriously threatened with a re-installment of the ancient, but now exploded doctrine of isopathy. At the present time, even, pepsin obtained from the stomachs of animals is seriously advocated in all forms of disorders affecting the human stomach; the use of ox-gall, obtained from the livers of cattle, is advocated as a sovereign remedy in many diseases peculiar to men with lame or crippled livers; the animal pancreas is likewise utilized to facilitate intestinal digestion, when this disorder is supposed to be dependent upon a lack of functional activity of that organ. Quite recently I listened to a paper read before a medical society, in which the ground was taken that dysentery was a disease due to the inactivity of both liver and pancreas, and cases were cited in which the exhibition of these remedies combined had sufficed to effect a cure. Of course the advantages to be gained from this line of treatment are obvious, but the principal good arises from metamorphoses taking place in the fats and starchy food products, rather than from any anti-septic properties which these agents are claimed to possess.

Failure of the digestive apparatus, however, is not always an indication for the employment of artificial ferments, as without a restricted dietary, these remedies prove of but temporary benefit. So unsatisfactory have been the results of this method in the hands of some physicians, that it has been abandoned. A physician made the remark to me lately, that he ordered digestive ferments only when all other remedies failed, and he did not know what else to give. My own experience with these preparations has been eminently satisfactory, but I have endeavored to exercise discretion in the selection of the dietary, when they were employed. Thus, in giving pepsin to assist a disabled stomach, the food should be selected with a view to lessen the work of the organ, while the exhibition of pancreatin requires that the demands upon intestinal digestion should be reduced to a minimum by excluding fats and starchy substances. Remarkable good results will often follow the adoption of a dietary regulated according to the foregoing indications, even without the exhibition of any form of medicine.

The truth is that too many people of this country are precipitating upon themselves a debilitated condition, with a class of diseases following in its train, that may be looked upon as certainly preventable, although but few practitioners have so far taken advantage of this practical fact. In nearly all diseases which have assumed a chronic character, the physician will seldom make a mistake in cutting off starchy food-products, while at the same time he supplies something which will assist in converting such articles that are permitted. To provide such a remedy, one which will be taken readily by persons whose stomachs have been made the dumping ground for the most diabolical combinations under the garb of medicine, is a requirement of no small magnitude.

Just here I may refer to diastase, a valuable principle found in malt extract, a remedial agent which has recently attracted considerable attention. Diastase is a soluble substance, and possesses the power of dissolving starch, convert it into gum (dextrin), and finally into grape sugar, of a substance, which upon analysis, closely resembles grape sugar as it exists naturally in the grape. The amylolytic properties of diastase are in some respects similar to that of the pancreatic juice, and when we desire to act upon starch alone, it will frequently prove serviceable when pancreatin cannot be used, owing to the destructive action of the gastric juice upon this delicate product. The activity of diastase is much like that of pepsin, except that the latter acts only upon albuminoids; the proteolytic power of a good pepsin is about the same as the amylolitic power of a good diastase, one part of the former being sufficient to convert two thousand parts of albumin into peptone, while one part of diastase will convert two thousand parts of starch into grape sugar.

In order to obtain some definite idea upon this subiect it will be advisable to consider the character of the more important food-products containing starch, as by eliminating these substances from the dietary, or restricting their use, patients will receive more benefit from the exhibition of malt extract and its contained diastase.

According to Professor Coleman, of Glasgow, Scotland, the following figures represent the proportion of starch contained in the food substances named:

| | | | | |
|---|---|---|---|---|
| Wheaten bread, | Percentage | of starch, | | 47.4 |
| Wheaten flour, | " | " | " | 66.3 |
| Oatmeal, | " | " | " | 58.4 |
| Potatoes, | " | " | " | 18.8 |
| Rice, | " | " | " | 79.1 |
| Arrow-Root, | " | " | " | 82.0 |

Starch, like sugar, fat, etc., is a heat producer, while albuminoids, such as fibrin, casein, albumin, etc., are flesh-formers. When too much starchy food is taken into the system, the amylolitic function is arrested, fermentations occur accompanied by more or less physical and mental depression, due to the local irritation set up and the absorption of noxious materials. The most casual observer cannot fail to understand the deleterious effects which must attend upon a disordered condition of the digestion such as that described, and the intelligent physician will readily apprecirte the benefits to be derived from the exhibition of remedies calculated to meet these difficulties. At one time bleeding was resorted to, and proving of temporary benefit, the plan was followed by a large number of practitioners; later, however, purgatives became the rule, but the more intelligent class of patients rebelled, and so they in turn have given place to antiseptics. Still without regulation of the diet, our practice under the new regime is exceedingly unsatisfactory. An ideal combination in this class of cases would include digestive ferments with the antiseptics, but owing to the incompatibility between the two, this plan cannot be adopted. Indeed, the physician too often finds that his resources have been exhausted, and is disposed to regret the uncertainty of all medicaments. This statement doubtless voices the sentiments of many physicians, and it is under such unfavorable auspices, I think, that attention should be given to malt extracts.

Physicians in special practice do not hesitate to order malt extract for patients by the dozen bottles, often without other medication, in cases which have been passed over to them by the general practitioner. My own experience with a reliable preparation has been so uniformly successful that I am prompted to publish my conclusions, believing that by this means others may be guided into the same channel. To determine what is meant by a reliable preparation, several questions are to be considered, the most important of which viewed from a medical standpoint, may be mentioned as follows:

1. Diastasic power, or its ability to convert starch.
2. Purity, or its freedom from substance calculated to impair the therapeutic value of the product.
3. Palatableness because we wish to avoid nauseating mixtures where malt extract is indicated.

These questions will be discussed in a general way, in the order of their occurrence. Attention first should be called to an erroneous impression which obtains, viz.—that malt extract, ale, beer and porter are substantially the same, and consequently some physicians are opposed to the use of either, believing it contrary to public policy to encourage the establishment of breweries. The facts are, that malt extract is a product which differs materially from all the others, in its manufacture, diastasic power and contained alcohol. Dr. Coleman's investigations form a specific contradiction of this assumption. Duly measured quantities of bread were placed in a watery alkaline solution, which was maintained at a body-heat for the period of six hours, with specified amounts of malt extract and the ordinary well-known English beverages, with the following results :

| | | | | |
|---|---|---|---|---|
| The Burton ale dissolved | 5 | per cent. | of the | starch. |
| The London porter " | 40 | " | " | " |
| The Wrexham ale " | 26 | " | " | " |
| Joh. Hoff's Malt Ext. | 60 | " | " | " |

The disastasic power, therefore, is a property which may be demonstrated, and in considering the claims of any preparation, this subject should receive our first attention. When this important quality is lacking, we are to enquire whether or not the cause is due to an excess of alcohol, or to the addition of salicylic acid or other objectionable substances as preventatives. Both alcohol in excess and salicylic acid retard and practically destroy the diastastic power of malt extract, which may account for the favorable results obtoined by Prof. Coleman with the genuine Joh. Hoff's malt extract, containing as it does, but three and one-half per cent. of alcohol and no salicylic acid. As much cannot be said of a malt extract, also called Hoff's, manufactured by Leopold Hoff, as repeated examinations by Professor Leffman, the well-known analytical chemist and expert, discovered the presence of a much larger proportion of alcohol and invariably salicylic acid. Prof. Leffman says: "The effects of salicylic acid have been extensively studied, and the unanimous opinion of sanitary chemists is, that it is very objectionable as an addition to any form of food or drink, and especially objectionable in malt extract. From some observations made in my own laboratory, it appears that not only does salicylic acid wholly suspend the action of diastase, to which malt owes its starch converting power, but that the starch digesting power of the pancreatic secretion is wholly suspended by it. It thus appears that the addition of this body is to render the extract not only inactive so far as its own function is concerned, but it introduces into the system an injurious substance, which interferes with another important function."

The preparation referred to by Prof. Leffman enjoys an enviable reputation on both sides of the Atlantic, having been the first product of the kind offered to the medical profession as early as 1847, and notwithstanding the attempts which have been made to supplant it, the fact remains that it is incomparably the best of all the numerous preparations now on the market as regards diastasic power, purity and palatableness. The improvements suggested by scientic study, together with long experience, have given the genuine Johann Hoff's article a reputation that has stimulated a host of imitators, but to-day it occupies a position far in advance of all competitors as an elegant, nutritious and efficient tonic, adapted alike to young and old, and especially to those in a debilitated condition dependent upon indiscretions in diet. It is an admirable remedy for the period of convalescence following long continued diseases, notably typhoid fever, when the functional activity of the intestinal glands is below par, and will be found of signal service in arresting the progress of all forms of disease in which failure of the digestive functions is a prominent factor.—*N. E. Med. Monthly.*

